# Access to antiretroviral therapy and survival in eastern Europe and central Asia: a case study in Armenia

**DOI:** 10.7448/IAS.17.1.18795

**Published:** 2014-08-04

**Authors:** Kylie-Ann Mallitt, Samvel R Grigoryan, Arshak S Papoyan, Handan C Wand, David P Wilson

**Affiliations:** 1 The Kirby Institute, University of New South Wales, Sydney, Australia; 2 National Center for AIDS Prevention, Yerevan, Armenia

**Keywords:** AIDS, survival, treatment, Europe

## Abstract

**Introduction:**

Antiretroviral therapy (ART) substantially improves the health of people living with HIV and contributes to preventing new infections. While HIV incidence is decreasing in most regions, the epidemic in eastern Europe continues to rise, as new infections currently outnumber the rate of ART initiation. In this study, we assess ART use in Armenia and its impact on the number of AIDS diagnoses and mortality.

**Methods:**

National surveillance data were obtained from the National Centre for AIDS Prevention, Armenia. Cox-proportional hazard models were used to determine the effect of demographic and clinical risk factors, including access to ART, on AIDS and mortality.

**Results:**

Among people diagnosed with HIV since 2005, approximately 40% per year were diagnosed with CD4<200 cells per mL. Overall, 232 people (57.1%) with AIDS or a low CD4 count had not received ART by the end of 2010. Mortality was 34.1% among people living with HIV who did not initiate ART, and 0.3% among people who received ART. Among people diagnosed with HIV from 1996 to 2010, age at diagnosis, no use of ART, likely mode of transmission, likely place of transmission, low baseline CD4 count and no STI diagnosis at last contact are significantly associated with death.

**Discussion:**

In Armenia, HIV is frequently diagnosed at a late stage of disease, indicating low testing rates. Of people diagnosed with HIV and in need of ART, a large proportion (approximately 60%) either do not provide consent for treatment, or are who migrants who cannot be located.

**Conclusions:**

Globally, the scale-up of ART has resulted in substantial reductions in mortality among individuals initiating therapy. However, in an era of momentum for treatment as prevention, treatment levels are not at adequate levels for preventing morbidities and mortality in some settings. Particular focus should be placed on key at-risk subgroups.

## Introduction

Eastern Europe and central Asia is the only region of the world where HIV incidence and AIDS-related deaths continue to increase [1–3]. From 2001 to 2010, the number of people living with HIV (PLHIV) in the region has risen by 250% [4]. The region is characterised by concentrated epidemics, predominantly among injecting drug users (IDUs) [1]. Rates of HIV are also high among other priority populations such as sex workers, men who have sex with men and migrant labourers [2]. Heterosexual transmission of HIV to the partners of IDUs and migrant workers is cause for concern that an epidemic in the region may become more generalised [5].

The increased burden of HIV in eastern Europe and central Asia is driven by poor access to antiretroviral therapy (ART) [2]. Combination ART first became available from 1996, with high coverage in developed countries [6]. A push for expanded access to ART in low- and middle-income countries occurred in early to mid-2000s, particularly with the World Health Organisations’ “3 by 5” initiative, which aimed to have three million people on ART by 2005 [7]. However, in practice, wide scale-up of ART access took place in these countries from 2006–2010, and in 2012, more than seven million PLHIVs had access to ART [6]. Universal coverage of timely ART among people in need reduces HIV-related morbidity and is emerging as a key prevention strategy [8,9]. However, in regions such as eastern Europe and central Asia, ART coverage has not achieved sufficient levels to sustain life among PLHIV [6].

In this study we analyse HIV and AIDS in the Republic of Armenia, a country representative of the epidemic in lower- and middle-income countries of eastern Europe and central Asia. Armenia has a population of three million people, with an estimated national HIV prevalence of 0.1% [2]. While most HIV infections in Armenia are attributable to heterosexual transmission, the HIV epidemic is also highly concentrated among IDUs with an estimated prevalence of 9.5% [2,10]. Armenia has a small, but rigorous and centralised, HIV/AIDS data collection system. ART first became available in Armenia in 2005 [10]. The aim of this paper is to assess the effect of ART coverage on AIDS diagnoses and mortality in the Republic of Armenia and to infer the adequacy of HIV testing rates.

## Methods

The number of people who had received ART each year since 2005 was extracted from surveillance data, and this was compared to the number of people who were eligible for ART. During the period of this study, eligibility for ART was defined in the Armenian National HIV/AIDS Treatment and Care Protocols as meeting at least one of the following criteria at diagnosis: a) the presence of clinical AIDS; b) CD4 count <200 cells per mL; or c) CD4 count <350 cells per mL if symptoms were present.

National HIV surveillance data were available from the National Center for AIDS Prevention (NCAP) in Armenia, from 1988 to 2010. NCAP is responsible for collecting all HIV testing data in Armenia, including outcomes from all processing of blood samples and the maintenance of a centralised national HIV surveillance database. These data include demographic and clinical information on people diagnosed with HIV, including age; sex; marital status; date of HIV, AIDS and death diagnoses; location of residence (marz, the highest administrative boundary); self-reported likely mode of transmission, self-reported place and date of likely transmission; presence of an sexually transmissible infection (STI); use of ART; and CD4 count at diagnosis. AIDS and STI diagnosis are reported at last contact. STI diagnoses were obtained by laboratory confirmation.

A retrospective cohort analysis of HIV and AIDS in the Republic of Armenia was conducted. Data from people diagnosed with HIV from January 1996 were used. Follow-up time was from date of HIV diagnosis to AIDS diagnosis, death or 31 December 2010. Demographic and clinical data are presented as frequencies and percentages for categorical data, or medians and interquartile ranges for continuous data. Cox proportional hazards modelling was used to determine whether these clinical and demographic risk factors were significantly associated with the development of AIDS and all-cause mortality, among people who are diagnosed with HIV in Armenia. Backwards stepwise modelling was used, and all variables which were significant at the *p*=0.2 level in univariate models were included in multivariate analyses. The threshold for significance in multivariate models was *p=*0.05. Age and sex were retained in the multivariate models regardless of statistical significance. The age group 20–29 was chosen as the reference category, as it is the youngest age group with sufficient numbers of PLHIV to ensure statistical precision of estimates. Kaplan-Meier curves were used to visualise the risk of death stratified by important significant variables. All statistical analyses were conducted in Stata v.12.

## Results

From 1996 to 2010 there were 968 reported cases of HIV, and 477 cases of AIDS. From 1996 to 2003, the number of HIV diagnoses in the Republic of Armenia remained low, at below 40 per year. From 2003 to present there has been a substantial rise in HIV diagnoses, with more than 140 cases diagnosed in 2010. The number of AIDS diagnoses in Armenia has followed a similar pattern. AIDS diagnoses remained at below 20 per year from 1996 to 2003, followed by an increase to 2010. Among PLHIV who develop AIDS, the median time between HIV diagnosis and AIDS diagnosis is 70 days, and for those who die, the median time to death after HIV diagnosis is 163 days.

Demographic and clinical characteristics of people diagnosed with HIV in Armenia (1996–2010), stratified by AIDS diagnosis and death, are shown in Table 1. The majority of people diagnosed with HIV were male (71.8%), aged 30–39 years (39.3%) and married (40.4%). The most common reported mode of HIV transmission was heterosexual contact (51.3%); and 39.7% were reported as IDUs. People who were diagnosed with AIDS (48.6%) or who died (53.2%) were disproportionately more likely to be IDUs. Only 25.2% of people diagnosed with HIV acquired HIV within Armenia, and the most common country of HIV acquisition was the Russian Federation (41.7%). Armenia's capital city of Yerevan was the most common location of residence at HIV diagnosis (42%), with other substantial numbers of cases in Shirak (11.1%), Lori (9.5%) and Armavir (9.0%). Among people diagnosed with HIV, 18.9% were also diagnosed with another STI. However, only 2.6% of people who died had been diagnosed with an STI.

**Table 1:** Demographic and clinical descriptive statistics (at diagnosis) by AIDS diagnosis and death (at last contact), among those diagnosed with HIV in Armenia (1996–2010)

Variable		Total, *n* (%)	Not diagnosed with AIDS, *n* (%)	Diagnosed with AIDS, *n* (%)	Alive, *n* (%)	Dead, *n* (%)
Total *n*		968	491	477	733	235
Age (years)	0–19	35 (3.6)	22 (4.5)	13 (2.7)	28 (3.8)	7 (3.0)
	20–29	275 (28.4)	175 (35.6)	100 (21.0)	238 (32.5)	37 (15.7)
	30–39	380 (39.3)	169 (34.4)	211 (44.2)	281 (38.3)	99 (42.1)
	40–49	218 (22.5)	97 (19.8)	121 (25.4)	145 (19.8)	73 (31.1)
	50+	60 (6.2)	28 (5.7)	32 (6.7)	41 (5.6)	19 (8.1)
Sex	Male	695 (71.8)	329 (67.0)	366 (76.7)	500 (68.2)	195 (83.0)
	Female	273 (28.2)	162 (33.0)	111 (23.3)	233 (31.8)	40 (17.0)
Marital status	Single	170 (17.6)	87 (17.7)	83 (17.4)	127 (17.3)	43 (18.3)
	Married	391 (40.4)	186 (37.9)	205 (43.0)	304 (41.5)	87 (37.0)
	Other	88 (9.0)	38 (7.7)	50 (10.5)	65 (8.9)	23 (9.8)
	Unknown	319 (33.0)	180 (36.7)	139 (29.1)	237 (32.3)	82 (34.9)
Mode of transmission	Heterosexual	497 (51.3)	286 (58.2)	211 (44.2)	404 (55.1)	93 (39.6)
	IDU	384 (39.7)	152 (31.0)	232 (48.6)	259 (35.3)	125 (53.2)
	Other^a^	37 (3.8)	21 (4.3)	16 (3.4)	28 (3.8)	9 (3.8)
	Unknown	50 (5.2)	32 (6.5)	18 (3.8)	42 (5.7)	8 (3.4)
Place of transmission	Armenia	244 (25.2)	134 (27.3)	110 (23.1)	198 (27.0)	46 (19.6)
	Russia	404 (41.7)	181 (36.9)	223 (46.8)	303 (41.3)	101 (43.0)
	Other	85 (8.8)	28 (5.7)	57 (11.9)	59 (8.0)	26 (11.1)
	Unknown	235 (24.3)	148 (30.1)	87 (18.2)	173 (23.6)	62 (26.4)
Location (marz)	Yerevan	407 (42.0)	189 (38.5)	218 (45.7)	301 (41.1)	106 (45.1)
	Other	551 (57.0)	292 (59.5)	259 (54.3)	423 (57.7)	128 (54.5)
	Unknown	10 (1.0)	10 (2.0)	0 (0.0)	9 (1.2)	1 (0.4)
Presence of an STI	No	784 (81.1)	430 (87.6)	354 (74.2)	555 (75.7)	229 (97.4)
	Yes	184 (18.9)	61 (12.4)	123 (25.8)	178 (24.3)	6 (2.6)
CD4 count at diagnosis	Median (IQR)	277 (87–497)	492 (378–647)	128 (42–269)	330 (127–515)	110 (39–281)

a Other modes of transmission include: men who have sex with men, children of mothers living with HIV and blood and organ recipients.

**Figure 1:**
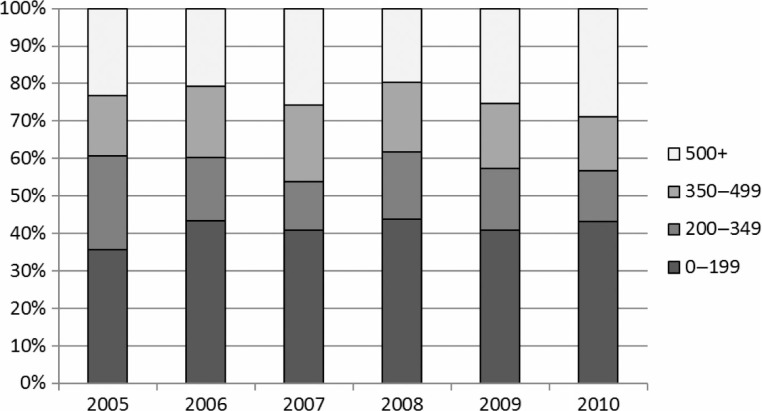
Baseline CD4 count (percentage) among people diagnosed with HIV in Armenia (2005–2010).

From 2005 (when ART became available in Armenia) to 2010, approximately 25% of people diagnosed with HIV had a baseline CD4 count of greater than 500 cells per mL. However, ~40% of people who are diagnosed with HIV each year had a baseline CD4 count of <200 cells per mL. These proportions were fairly consistent from 2005 to 2010 (Figure 1). Prior to 2005, 101 people (35.3% of HIV diagnoses) had a baseline CD4 count recorded. From 2005 to 2010, this figure rose to 535 people (78.5% of HIV diagnoses). Since 2005, the median CD4 count among was 121.5 cells per mL (IQR 238) among people who were eligible for ART, and 503 (IQR 283) for those who were not eligible.

Table 2 shows the number of people who met one of the three eligibility criteria for ART (used during the period of this study) in each year from 2005 to 2010 and indicates how many of these eligible people received ART to the end of 2010. Among those diagnosed with HIV since 2005, 232 eligible people (57.1%) had not received ART by the end of 2010. This ranges from 64.3% in 2005 to 50% in 2008.

**Table 2:** Number of people diagnosed with HIV (2005–2010) in Armenia who were “eligible”^a^ for ART, by whether they had received ART to either the end of 2010 or to time of death

	Eligible^a^, *n* (%)			Ineligible^a^, *n* (%)				
	
Year of Diagnosis	Received ART^b^	Never received ART	Total	Received ART^b^	Never received ART	Total	Diagnoses, *n*	Deaths^b^, *n*
2005	15 (35.7)	27 (64.3)	42 (100)	1 (3.0)	32 (97.0)	33 (100)	75	29
2006	19 (54.3)	16 (45.7)	35 (100)	3 (9.4)	29 (90.6)	32 (100)	67	24
2007	29 (44.6)	36 (55.4)	65 (100)	5 (11.9)	37 (88.1)	42 (100)	107	26
2008	38 (50.0)	38 (50.0)	76 (100)	4 (6.7)	56 (93.3)	60 (100)	136	36
2009	38 (39.2)	59 (60.8)	97 (100)	7 (13.5)	45 (86.5)	52 (100)	149	19
2010	35 (38.5)	56 (61.5)	91 (100)	3 (5.3)	54 (94.7)	57 (100)	148	19
Overall	174 (42.9)	232 (57.1)	406 (100)	23 (8.3)	253 (91.7)	276 (100)	682	153

a Persons eligible for ART meet at least one of the following at diagnosis: a) the presence of clinical AIDS; b) CD4 count <200/µL; or c) CD4 count <350/µL if symptoms are present.

b To the end of December, 2010.

Age (*p=*0.002) and baseline CD4 count (*p*<0.001) were significantly associated with the development of AIDS (Table 3). For every 100 cells per mL increase in CD4 at diagnosis, PLHIV were 0.57 times less likely to develop AIDS (95% CI 0.53–0.61).

**Table 3:** Cox proportional hazards survival analysis of the effect of clinical and demographic risk factors on time to AIDS and time to death among PLHIV in Armenia (1996–2010)

Outcome	Explanatory variable	Level	Univariate hazard ratio (95% CI)	Univariate *p*-value	Multivariate hazard ratio (95% CI)	Multivariate *p*-value
Time to AIDS	Age (in years)	0–19	1.11 (0.62–1.98)		1.73 (0.93–3.25)	
diagnosis	20–29	1	<0.001	1	0.002
	30–39	1.93 (1.52–2.45)		1.40 (1.07–1.81)	
	40–49	1.98 (1.52–2.58)		1.71 (1.28–2.30)	
	50+	2.18 (1.46–3.25)		2.04 (1.30–3.19)	
	Sex	Male	1	0.001		
	Female	0.76 (0.61–1.93)			
	Marital status	Single	1	0.042		
	Married	1.27 (0.98–1.63)			
	Other/unknown	1.00 (0.77–1.30)			
	Likely mode of transmission	Sexual contact	1	<0.001		
	IDU	1.45 (1.20–1.74)			
		Other/unknown	0.88 (0.61–1.26)			
	Likely place of transmission	Armenia	1	<0.001		
	Russia	1.26 (1.00–1.58)			
		Other/Unknown	0.75 (0.58–0.97)			
	Place of residence	Yerevan	1	0.329		
		Other	0.91 (0.76–1.10)			
	STI	No	1	<0.001		
		Yes	1.74 (1.42–2.14)			
	Baseline CD4	Per 100 cells/mL	0.58 (0.55–0.62)	<0.001	0.59 (0.55–0.63)	<0.001
Time to death	Age (in years)	0–19	1.50 (0.67–3.37)		3.63 (0.99–13.4)	
	20–29	1	<0.001	1	0.002
	30–39	2.25 (1.54–3.28)		1.30 (0.76–2.23)	
	40–49	3.23 (2.17–4.80)		2.25 (1.27–3.99)	
	50+	3.65 (2.09–6.37)		3.60 (1.50–8.65)	
	Sex	Male	1	<0.001		
	Female	0.52 (0.37–0.73)			
	Marital status	Single	1	0.910		
	Married	0.84 (0.65–1.34)			
	Other/unknown	0.98 (0.67–1.40)			
	Likely mode of	Sexual contact	1	<0.001	1	0.040
	transmission	IDU	1.70 (1.30–2.22)		1.61 (1.06–2.43)	
		Other/unknown	1.01 (0.60–1.70)		0.60 (0.19–1.91)	
	Likely place of	Armenia	1	0.242	1	0.016
	transmission	Russia	1.32 (0.93–1.87)		0.80 (0.49–1.32)	
		Other/unknown	1.10 (0.77–1.59)		0.45 (0.25–0.80)	
	Place of residence	Yerevan	1	0.808		
		Other	1.03 (0.80–1.34)			
	STI	No	1	<0.001	1	0.045
		Yes	0.10 (0.05–0.23)		0.42 (0.18–0.98)	
	Baseline CD4	Per 100 cells/mL	0.72 (0.65–0.79)	<0.001	0.61 (0.55–0.69)	<0.001
	ART use	No	1	<0.001	1	<0.001
		Yes	0.01 (0.00–0.06)		0.01 (0.00–0.05)	

**Figure 2:**
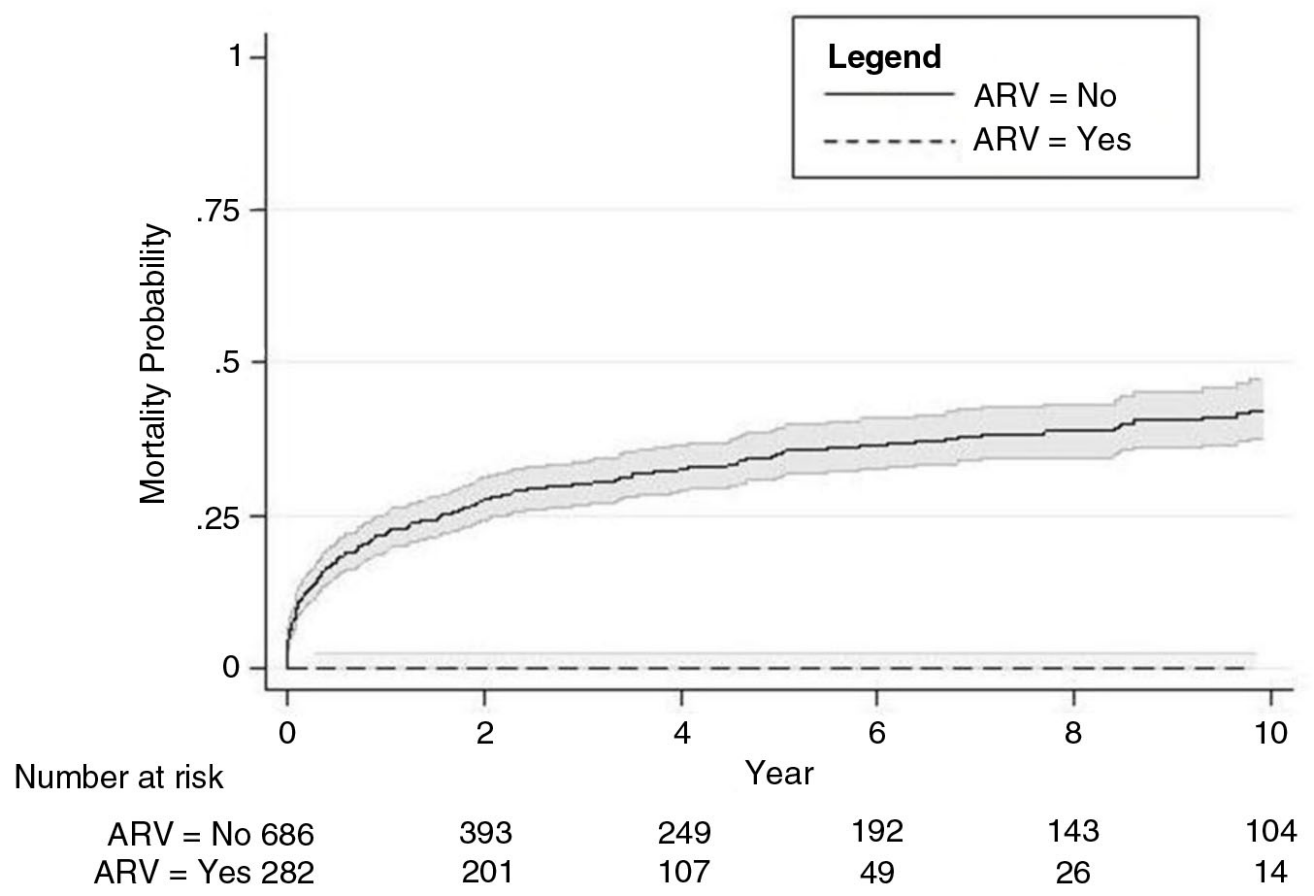
Risk of mortality after HIV diagnosis in Armenia, stratified by ART use (1996–2010).

Age (*p=*0.002), likely mode of transmission (0.040), likely place of transmission (0.016), no use of ART (*p*<0.001), baseline CD4 count (*p*<0.001) and STI diagnosis (*p*=0.045) were significantly associated with death among people diagnosed with HIV (1996–2010) (Table 3). ART use was associated with 99% lower risk of mortality than those who did not have access to ART (95% CI 0.00–0.05). The survival curve for the hazard of death stratified by ART use is shown in Figure 2. Among the 282 people who received ART, one person (0.3%) died; while mortality was 34.1% among people who did not receive ART (234 out of 686 people). The presence of an STI was associated with a reduction in all-cause mortality (HR=0.42; 95% CI: 0.18–0.98).

## Discussion

The rate of mortality among people diagnosed with HIV in Armenia (1996–2010) is very high, and overall there is low coverage of ART. It can be inferred that testing rates are also low, as there is a consistently low median CD4 count at diagnosis over time. People who receive ART are 99% less likely to die than people who do not receive ART.

Among people diagnosed with HIV from 1996 to 2010, age and baseline CD4 counts were significantly associated with the development of AIDS. Age, mode of transmission, place of transmission, no use of ART, low baseline CD4 count and no STI diagnosis are significantly associated with death. It is likely that the high mortality among people without a clinical diagnosis of an STI is associated with a high rate of undiagnosed STIs. People who have STIs that are more engaged with the health system are more likely to be diagnosed early with HIV and treated appropriately. A limitation of this data is that there may be loss to follow-up, resulting in incomplete data on AIDS and mortality. However, the observed number of AIDS diagnoses and deaths are likely to be an underestimate of the true number. ART use was not included in the multivariate analysis of risk factors for AIDS because in Armenia approximately 50% of people received ART concurrently with their AIDS diagnosis, and 50% received ART after their AIDS diagnosis.

From 2005 to 2010, median CD4 count at diagnosis has remained low, at 273 per mL. These data indicate that people are typically being diagnosed with HIV in Armenia several years after initial HIV infection. People diagnosed with HIV who suffer severe adverse health outcomes (AIDS diagnosis and death) are at an advanced stage of disease at the time of HIV diagnosis. Based on evidence from another country in the region, this is likely associated with low testing rates due to clinical, social and demographic factors such as comorbidities, poor access to health services and stigma [11]. Routine HIV testing among pregnant women in Armenia is very high [10]. However, testing rates among key at-risk subpopulations, such as IDUs and migrant workers, must be urgently addressed.

Individuals who are younger or older than 20–29 years at diagnosis have the highest mortality. This age group has the lowest testing rate in Armenia, and may reflect the fact that these individuals have poor engagement with the health-care system, and poorer follow-up. However, men of this age may also be more likely to be seasonal workers less, and thus less likely to receive treatment. The percentage of IDUs who received an HIV test in the past 12 months and who know the results was 20% [12]. Seasonal migrant workers, particularly to higher prevalence countries such as Ukraine and Russia, are very common in Armenia with a general population HIV prevalence of 1.6 and 1.1%, respectively [1,2,13]. Migration can result in higher levels of commercial and casual sex [5,11]. Armenian women married to migrants are significantly more likely to be diagnosed with an STI than those married to non-migrants [13]. In the absence of additional testing and treatment among these subpopulations, the HIV epidemic in Armenia is expected to rise [1].

In all low- and middle-income countries globally, the average coverage of ART among those in need was 47% in 2010 and only 22% in low and middle-income countries in eastern Europe and central Asia [2,14]. This is exacerbated among vulnerable groups who remain more isolated from mainstream public health services [11]. The majority of PLHIV in the region are IDUs; however, they make up less than 25% of people on ART [14]. HIV testing rates in eastern Europe and central Asia are also very low [2]. Among people diagnosed with HIV since 2005 in Armenia, 62.8% of people with AIDS or a low CD4 count had not received ART by the end of 2010. Armenia has reported achieving universal access to ART at the end of 2007, offering treatment to at least 80% of patients in need; and 100% coverage to PLHIV in need from 2007 to 2009 [15,16]. However, these figures do not include a large proportion (approximately 60%) of PLHIV who meet the clinical criteria for ART access, but who either do not provide consent for treatment, or who are migrants who cannot be located (S. Grigoryan, National AIDS Center Armenia, personal communication, 2 November, 2011). Priorities for clinical care of PLHIV must encompass all aspects of the treatment cascade where continuity is maintained through HIV diagnosis, linkage to care, retention in care, ART access and treatment adherence with suppressed virus [17]. The percentage of PLHIV who are eligible for treatment, but who are not on ART, should be associated with a lack of access to ART. “Lack of access” is a broad term that encompasses not only whether a patient is offered ART in a clinic, but also the capacity of records and information systems to track patients if they relocate and ensuring patients are referred and entered into an alternative ARV clinic, the social pressures and stigmas that may make ART more or less desirable, and perceptions of other treatment options. These are all access issues that require addressing in order to translate universal offering of ART to true universal coverage of ART. In Armenia, a framework for domestic priorities has been established. The National Strategic Plan on the Response to the HIV Epidemic in the Republic of Armenia for 2012–2016 [18] has been developed. Importantly, a higher threshold of ART initiation has been accepted (CD4 <350), as recommended by WHO guidelines [19].

## Conclusions

Up to 2010, HIV-related mortality was high and ART coverage was low. HIV treatment must be up-scaled in the Republic of Armenia, and more broadly in eastern Europe and central Asia, to halt the mortality and morbidity associated with HIV. This would also have the secondary benefit of reducing the spread of infection to others. Particular focus should be placed on key at-risk subgroups, such as IDUs and migrant workers.
